# Whole-genome sequencing analysis of clinical carbapenemase-producing bacteria isolated in Dakar, Senegal

**DOI:** 10.1128/spectrum.03704-25

**Published:** 2026-07-16

**Authors:** Moustoifa Ibrahim, Cheikh Ibrahima Lo, Fatoumata Diallo, Komla Mawunyo Dossouvi, Ousmane Sadio, Adja Bousso Gueye, Moussa Moise Diagne, Safiétou Sanghe, Madiagne Der, Yakhya Dieye, Mouhamadou Lamine Dia

**Affiliations:** 1 Department of Animal Biology, Faculty of Science and Technology, Cheikh Anta Diop University of Dakar 89230, Dakar, Senegal; 2 Microbiology pole Institut Pasteur de Dakar, Dakar, Senegal; 3 Laboratory of Bacteriology-Virology, National University Hospital Center of Fann, Dakar, Senegal; 4 Department of Microbiology and Genomics, Global Health Research Institute of Lomé 847959 , Lomé, Togo; 5 Virology pole Institut Pasteur de Dakar, Dakar, Senegal; 6 Faculty of Medicine, Pharmacy, and Dentistry, Cheikh Anta Diop University of Dakar 89230, Dakar, Senegal; JMI Laboratories, North Liberty, lowa, USA

**Keywords:** antimicrobial resistance, carbapenem-resistant bacteria, genomic surveillance, Africa, *VEB-6*

## Abstract

Antimicrobial resistance, particularly to carbapenems, represents a major global public health threat. In Senegal, the increasing prevalence of carbapenem-resistant Gram-negative bacilli compromises the management of severe infections. This study, conducted between October 2022 and March 2024 at the Centre Hospitalier National Universitaire de Fann and the Institut Pasteur de Dakar, investigated 50 carbapenem-resistant bacterial isolates. Species identification was performed using Vitek 2 and MALDI-TOF MS, and antimicrobial susceptibility was assessed by disk diffusion testing. Whole-genome sequencing enabled the detection of resistance genes, plasmid replicons, and clonal lineages through MLST analysis. Among the 50 isolates, 35 (70%) carried at least one carbapenemase gene. The *bla_NDM-5_* gene was the most frequent (15/35; 42.9%), followed by *bla*_*OXA-181*_ (20.0%), *bla_NDM-1_* (14.3%), and *bla_OXA-484_* (11.4%). Co-occurrences of carbapenemase genes were observed (17.1%), including *bla_NDM-5_ + blaOXA_OXA-181_, bla_OXA-23_ + blaOXA_OXA-68_,* and *bla_OXA-70_ + blaOXA_OXA-420_*. These isolates extensively co-harbored other resistance determinants, most notably the *bla_CTX-M-15_* extended-spectrum β-lactamase (ESBL) gene. Genes conferring high-level resistance to aminoglycosides (*rmtB, armA*) and quinolones [*aac(6′)-Ib-cr, qnrA, qnrB*] were also identified. The predominant plasmid types were IncF (91.4%) and Col (62.9%). MLST analysis showed that, in this study collection (*n* = 35), *Escherichia coli* ST410 (7/35; 20.0%) and ST648 (3/35; 8.6%), as well as *Klebsiella pneumoniae* ST307 (3/35; 8.6%) and ST147 (2/35; 5.7%), were the most frequently detected sequence types. Notably, this study reports the first detection of the *bla*_*VEB-6*_ gene in Senegal. These findings highlight the circulation of multidrug-resistant clones harboring carbapenemase genes and underscore the urgent need to strengthen surveillance and infection control measures in Senegalese hospitals.

**IMPORTANCE:**

Carbapenem resistance among Gram-negative bacilli represents a global public health emergency, particularly in resource-limited settings where therapeutic options are scarce. In Senegal, data on the genetic mechanisms underlying this resistance remain limited. This study is among the few that have employed whole-genome sequencing to characterize carbapenemase-producing bacteria. The identification of high-risk clones, such as *Escherichia coli* ST410 and *Klebsiella pneumoniae* ST307, along with the first detection of the *bla*_*VEB-6*_ gene in Senegal, highlights the local spread of multidrug-resistant lineages. By combining phenotypic and genomic analyses, this research provides essential insights to strengthen genomic surveillance, optimize antibiotic use, and improve infection prevention and control strategies in Senegalese hospitals.

## INTRODUCTION

Antibiotic resistance among Gram-negative bacilli (GNB) represents a significant public health threat. The World Health Organization classifies carbapenem-resistant bacteria as critical priority pathogens due to their high mortality rates and limited therapeutic options (1). In 2021, these resistances contributed to over one million associated deaths, including more than 200,000 directly attributable to carbapenem resistance (2). Such infections, often acquired in healthcare settings, prolong hospital stays, and increase treatment costs (3).

The main mechanisms of resistance in GNB rely on the production of carbapenemases, enzymes capable of inactivating carbapenems. The most frequently reported carbapenemases include KPC (class A), NDM, VIM, IMP (class B metallo-β-lactamases), and OXA-48-like enzymes (class D) (4, 5). Carbapenemases often coexist with other resistance genes, including those encoding extended-spectrum β-lactamases (ESBLs) and genes conferring resistance to aminoglycosides and quinolones (6), generating highly multidrug-resistant profiles and further limiting treatment options.

The dissemination of *bla_NDM_* genes and *bla_OXA_* genes encoding carbapenemases (notably OXA-48-like) is largely mediated by mobile genetic elements, such as plasmids and transposons, facilitating their horizontal transfer between different bacterial species (7–9). Among the plasmids involved in the spread of these genes, replicons of the IncF, IncX3, IncL/M, IncH, and IncC types have been frequently reported in the literature (9). The coexistence of multiple plasmid types within a single strain promotes the accumulation and simultaneous dissemination of multiple resistance determinants (9). In addition, certain clonal lineages associated with these genes, such as *Escherichia coli* ST410, ST167, and ST131, as well as *Klebsiella pneumoniae* ST147 and ST307, have been reported to be involved in the international spread of NDM- and OXA-type carbapenemases, contributing to the emergence and dissemination of multidrug-resistant strains (10–12).

In Senegal, although carbapenem resistance has been reported in several hospitals, genomic sequencing data remain very limited (13). This lack of detailed information on clonal diversity, plasmids, and resistance genes hinders a comprehensive understanding of the circulation of multidrug-resistant strains. In this context, the present study was undertaken to better characterize carbapenem-resistant GNB and provide essential data to strengthen national surveillance and control efforts.

## RESULTS

### Characteristics of the study population

A total of 50 isolates were sequenced in this study. Among them, 35 were confirmed as carbapenemase-producing strains. The analyses presented below focus exclusively on these 35 isolates.

A male predominance was observed among the patients carrying these isolates, representing 54.29% of the study population. The mean age of the patients was 57.63 years (range: 14–87 years). The majority of patients were hospitalized at the time of sampling (62.86%). With regard to the type of specimens analyzed, blood cultures were the most frequently recovered (31%), followed by pus samples (29%) and urine samples (26%) (Table 1).

**TABLE 1:** Demographic and clinical characteristics of patients from whom the strains were isolated (*n* = 35)^*a*^

Variables	Categories	Number (*n* = 35)	Percentage (%)
Sex	Male	19	54.29
	Female	16	45.71
Age (years)	[14–34]	5	14.29
	[35–55]	5	14.29
	[56–76]	20	57.13
	≥77	5	14.29
Mean age (years)	57.63		
Median (years)	59		
Patient status	Hospitalized	22	62.86
	Non-hospitalized	13	37.14
Sample type	Blood	11	31
	Pus	10	29
	Urine	9	26
	Bronchial aspirate (LAB)	3	9
	Sputum	1	3
	Vaginal swab (PS)	1	3

^*a*^ LAB, bronchial aspirate; PS, vaginal swab.

### Characteristics of the isolates

#### Distribution of carbapenemase-producing isolates by bacterial species

Of the 35 carbapenemase-producing isolates, the species distribution was as follows: *E. coli* (49%), *K. pneumoniae* (31%), *Acinetobacter baumannii* (9%), *Proteus mirabilis* (6%), and *Enterobacter hormaechei* (6%) (Table 3).

### Antibiotic resistance profile of the isolated strains

Resistance to carbapenems was very high (ertapenem 100%, imipenem 97%), as was resistance to quinolones (100%). Antibiotic susceptibility testing revealed high resistance rates to penicillins and penicillin-beta-lactamase inhibitors (100% for ampicillin and amoxicillin-clavulanic acid, 100% for ticarcillin) and cephalosporins, with 100% resistance to first-generation cephalosporins, 100% to second-generation cephalosporins, and 97%–100% to third-generation cephalosporins (cefotaxime and ceftazidime). Only aminoglycosides, particularly amikacin, showed notable activity of 77% (Table 2).

**TABLE 2:** Antibiotic resistance profile of the bacterial strains^*a*^^,*b*,*c*^

Biosample ID	Species	ST	ERT	IMP	CAZ	TPZ	AMC	TIC	FOX	CTX	CF	AMP	AK	GEN	TOB	NA	LVX	CIP	OFX	NIT	SXT
SAMN47862014	*E. coli*	410	**R**	**R**	**R**	**R**	**R**	**R**	**R**	**R**	**R**	**R**	S	**R**	**R**	**R**	**R**	**R**	**R**	S	**R**
SAMN47862015	*E. coli*	410	**R**	**R**	**R**	**R**	**R**	**R**	**R**	**R**	**R**	**R**	S	**R**	**R**	**R**	**R**	**R**	**R**	S	**R**
SAMN47862022	*E. coli*	410	**R**	**R**	**R**	**R**	**R**	**R**	**R**	**R**	**R**	**R**	S	S	S	**R**	**R**	**R**	**R**	S	**R**
SAMN47862027	*E. coli*	410	**R**	**R**	**R**	**R**	**R**	**R**	**R**	**R**	**R**	**R**	S	S	S	**R**	**R**	**R**	**R**	S	**R**
SAMN47862028	*E. coli*	410	**R**	**R**	**R**	**R**	**R**	**R**	**R**	**R**	**R**	**R**	S	S	S	**R**	**R**	**R**	**R**	S	**R**
SAMN47862031	*E. coli*	410	**R**	**R**	**R**	**R**	**R**	**R**	**R**	**R**	**R**	**R**	**R**	**R**	**R**	**R**	**R**	**R**	**R**	**R**	**R**
SAMN47862033	*E. coli*	410	**R**	**R**	**R**	**R**	**R**	**R**	**R**	**R**	**R**	**R**	S	S	S	**R**	**R**	**R**	**R**	**R**	**R**
SAMN47862003	*E. coli*	648	**R**	**R**	**R**	**R**	**R**	**R**	**R**	**R**	**R**	**R**	S	**R**	**R**	**R**	**R**	**R**	**R**	S	S
SAMN47862010	*E. coli*	648	**R**	**R**	**R**	**R**	**R**	**R**	**R**	**R**	**R**	**R**	S	**R**	**R**	**R**	**R**	**R**	**R**	S	**R**
SAMN47862011	*E. coli*	648	**R**	**R**	**R**	**R**	**R**	**R**	**R**	**R**	**R**	**R**	S	**R**	**R**	**R**	**R**	**R**	**R**	S	**R**
SAMN47862029	*E. coli*	90	**R**	**R**	**R**	**R**	**R**	**R**	**R**	**R**	**R**	**R**	**R**	**R**	**R**	**R**	**R**	**R**	**R**	**R**	**R**
SAMN47862034	*E. coli*	44	**R**	**R**	**R**	**R**	**R**	**R**	**R**	**R**	**R**	**R**	S	**R**	**R**	**R**	**R**	**R**	**R**	S	**R**
SAMN47862035	*E. coli*	10	**R**	S	**R**	**R**	**R**	**R**	**R**	**R**	**R**	**R**	S	S	S	**R**	**R**	**R**	**R**	**R**	**R**
SAMN47862004	*E. coli*	361	**R**	**R**	**R**	**R**	**R**	**R**	**R**	**R**	**R**	**R**	S	S	S	**R**	**R**	**R**	**R**	S	**R**
SAMN47862007	*E. coli*	405	**R**	**R**	**R**	**R**	**R**	**R**	**R**	**R**	**R**	**R**	S	S	S	**R**	**R**	**R**	**R**	S	**R**
SAMN47862032	*E. coli*	167	**R**	**R**	**R**	**R**	**R**	**R**	**R**	**R**	**R**	**R**	**R**	**R**	**R**	**R**	**R**	**R**	**R**	**R**	**R**
SAMN47862030	*E. coli*	4981	**R**	**R**	**R**	**R**	**R**	**R**	**R**	**R**	**R**	**R**	**R**	**R**	**R**	**R**	**R**	**R**	**R**	**R**	**R**
SAMN47862005	*K. pneumoniae*	307	**R**	**R**	**R**	**R**	**R**	**R**	**R**	**R**	**R**	**R**	S	**R**	**R**	**R**	**R**	**R**	**R**	**R**	**R**
SAMN47862013	*K. pneumoniae*	307	**R**	**R**	**R**	**R**	**R**	**R**	**R**	**R**	**R**	**R**	S	**R**	**R**	**R**	**R**	**R**	**R**	S	**R**
SAMN47862023	*K. pneumoniae*	307	**R**	**R**	**R**	**R**	**R**	**R**	**R**	**R**	**R**	**R**	S	**R**	**R**	**R**	**R**	**R**	**R**	**R**	**R**
SAMN47862019	*K. pneumoniae*	147	**R**	**R**	**R**	**R**	**R**	**R**	**R**	**R**	**R**	**R**	**R**	**R**	**R**	**R**	**R**	**R**	**R**	**R**	**R**
SAMN47862020	*K. pneumoniae*	147	**R**	**R**	**R**	**R**	**R**	**R**	**R**	**R**	**R**	**R**	S	S	S	**R**	**R**	**R**	**R**	**R**	**R**
SAMN47862018	*K. pneumoniae*	323	**R**	**R**	**R**	**R**	**R**	**R**	**R**	**R**	**R**	**R**	S	**R**	**R**	**R**	**R**	**R**	**R**	**R**	**R**
SAMN47862024	*K. pneumoniae*	323	**R**	**R**	**R**	**R**	**R**	**R**	**R**	**R**	**R**	**R**	S	**R**	**R**	**R**	**R**	**R**	**R**	**R**	**R**
SAMN47862006	*K. pneumoniae*	ND	**R**	**R**	**R**	**R**	**R**	**R**	**R**	**R**	**R**	**R**	**R**	**R**	**R**	**R**	**R**	**R**	**R**	**R**	**R**
SAMN47862016	*K. pneumoniae*	ND	**R**	**R**	**R**	**R**	**R**	**R**	**R**	**R**	**R**	**R**	S	**R**	**R**	**R**	**R**	**R**	**R**	**R**	**R**
SAMN47862012	*K. pneumoniae*	101	**R**	**R**	**R**	**R**	**R**	**R**	**R**	**R**	**R**	**R**	S	**R**	**R**	**R**	**R**	**R**	**R**	**R**	**R**
SAMN47862036	*K. pneumoniae*	25	**R**	**R**	**R**	**R**	**R**	**R**	**R**	**R**	**R**	**R**	**R**	**R**	**R**	**R**	**R**	**R**	**R**	**R**	**R**
SAMN47862021	*A. baumannii*	642	**R**	**R**	S	**R**	**R**	**R**	**R**	**R**	**R**	**R**	S	**R**	**R**	**R**	**R**	**R**	**R**	**R**	**R**
SAMN47862026	*A. baumannii*	23	**R**	**R**	**R**	**R**	**R**	**R**	**R**	**R**	**R**	**R**	S	S	S	**R**	**R**	**R**	**R**	**R**	S
SAMN47862017	*A. baumannii*	ND	**R**	**R**	**R**	**R**	**R**	**R**	**R**	**R**	**R**	**R**	S	**R**	**R**	**R**	**R**	**R**	**R**	**R**	**R**
SAMN47862025	*P. mirabilis*	ND	**R**	**R**	**R**	**R**	**R**	**R**	**R**	**R**	**R**	**R**	S	**R**	**R**	**R**	**R**	**R**	**R**	**R**	**R**
SAMN47862037	*P. mirabilis*	ND	**R**	**R**	**R**	**R**	**R**	**R**	**R**	**R**	**R**	**R**	**R**	**R**	**R**	**R**	**R**	**R**	**R**	**R**	**R**
SAMN47862008	*E. hormaechei*	171	**R**	**R**	**R**	**R**	**R**	**R**	**R**	**R**	**R**	**R**	S	S	S	**R**	**R**	**R**	**R**	**R**	S
SAMN47862009	*E. hormaechei*	121	**R**	**R**	**R**	**R**	**R**	**R**	**R**	**R**	**R**	**R**	S	**R**	**R**	**R**	**R**	**R**	**R**	**R**	**R**
**% Resistant**			**100**	**97**	**97**	**100**	**100**	**100**	**100**	**100**	**100**	**100**	**23**	**71**	**71**	**100**	**100**	**100**	**100**	**66**	**91**

^*a*^ R indicates resistant; S indicates susceptible.

^*b*^ ERT, ertapenem; IMP, imipenem; CAZ, ceftazidime; TPZ, piperacillin/tazobactam; AMC, amoxicillin/clavulanic acid; TIC, ticarcillin; FOX, cefoxitin; CTX, cefotaxime; CF, cephalothin; AMP, ampicillin; AK, amikacin; GEN, gentamicin; TOB, tobramycin; NA, nalidixic acid; LVX, levofloxacin; CIP, ciprofloxacin; OFX, ofloxacin; NIT, nitrofurantoin; SXT, trimethoprim/sulfamethoxazole.

^*c*^ Gray shading and boldface "R" indicate resistant isolates.

### Distribution of sequence types by species

Multiple sequence types (STs) were observed among the isolates included in this study. In *E. coli*, ST410 and ST648 were the most prevalent, while in *K. pneumoniae*, ST307 and ST147 predominated, followed by ST323. Other STs were encountered occasionally. In total, five isolates corresponded to untyped MLST profiles (ND) (Table 3).

**TABLE 3:** Distribution of antimicrobial resistance (AMR) genes, plasmids, and STs among the bacterial isolates studied^*a*^^,*c*^

Biosample ID	Species	ST	*n* (%)	Carbapenemases—NDM			Carbapenemases—OXA							ESBL					Aminoglycosides							Quinolones				
				NDM-5	NDM-1	NDM-15	OXA-181	OXA-484	OXA-23	OXA-48	OXA-68	OXA-70	OXA-420	CTX-M-15	TEM	CMY	SHV	VEB-6	aac(6′)-Ib-cr	aph(6)-Id	aph(3″)-Ib	aac(3)-IIa	aadA2	rmtB	armA	qnrB	qnrS1	qnrA	qepA4	Replicon
SAMN47862014	*E. coli*	410	7 (41%)	Yes	No	No	Yes	No	No	No	No	No	No	Yes	Yes	Yes	No	No	Yes	Yes	No	No	No	No	No	No	No	Yes	No	IncF, col
SAMN47862015	*E. coli*	410		Yes	No	No	Yes	No	No	No	No	No	No	Yes	Yes	Yes	No	No	Yes	Yes	Yes	No	No	No	No	No	No	No	No	IncF, col
SAMN47862022	*E. coli*	410		No	No	No	No	Yes	No	No	No	No	No	Yes	No	Yes	No	No	No	Yes	Yes	No	Yes	No	No	Yes	Yes	No	Yes	IncF, IncI, col
SAMN47862027	*E. coli*	410		No	No	No	No	Yes	No	No	No	No	No	No	No	Yes	No	No	No	No	No	No	No	No	No	No	Yes	No	Yes	IncF
SAMN47862028	*E. coli*	410		Yes	No	No	No	No	No	No	No	No	No	Yes	No	No	No	No	No	No	No	No	No	No	No	No	No	No	No	IncF
SAMN47862031	*E. coli*	410		Yes	No	No	No	No	No	No	No	No	No	Yes	Yes	No	No	No	No	No	No	No	No	Yes	No	No	No	No	No	IncF, col
SAMN47862033	*E. coli*	410		No	No	No	No	Yes	No	No	No	No	No	No	No	Yes	No	No	No	No	No	No	Yes	No	No	No	No	No	Yes	IncF, col
SAMN47862003	*E. coli*	648	3 (18%)	Yes	No	No	No	No	No	No	No	No	No	Yes	Yes	Yes	No	No	Yes	No	No	Yes	Yes	No	No	No	No	No	No	IncF, col
SAMN47862010	*E. coli*	648		Yes	No	No	No	No	No	No	No	No	No	Yes	Yes	Yes	No	No	Yes	No	No	Yes	Yes	No	No	No	No	No	No	IncF, IncI, col
SAMN47862011	*E. coli*	648		Yes	No	No	No	No	No	No	No	No	No	Yes	Yes	Yes	No	No	Yes	No	No	Yes	Yes	No	No	No	No	No	No	IncF, IncI, col
SAMN47862029	*E. coli*	90	1 (6%)	Yes	No	No	No	No	No	No	No	No	No	No	Yes	No	No	No	Yes	Yes	No	No	No	Yes	No	No	Yes	No	No	IncF, col
SAMN47862034	*E. coli*	44	1 (6%)	Yes	No	No	Yes	No	No	No	No	No	No	Yes	Yes	Yes	No	No	Yes	No	No	No	No	No	No	No	Yes	No	No	IncF, col
SAMN47862035	*E. coli*	10	1 (6%)	No	No	No	Yes	No	No	No	No	No	No	No	Yes	No	No	No	No	Yes	Yes	No	No	No	No	No	Yes	No	No	IncF, col
SAMN47862004	*E. coli*	361	1 (6%)	Yes	No	No	No	No	No	No	No	No	No	Yes	No	Yes	No	No	No	No	No	No	Yes	No	No	No	No	No	No	IncF, IncI
SAMN47862007	*E. coli*	405	1 (6%)	Yes	No	No	No	No	No	No	No	No	No	No	No	Yes	No	No	No	No	No	No	Yes	No	No	No	No	No	No	IncF, IncI
SAMN47862032	*E. coli*	167	1 (6%)	No	No	No	No	Yes	No	No	No	No	No	No	No	Yes	No	No	No	No	No	No	No	No	No	No	Yes	No	No	IncF, IncI, col
SAMN47862030	*E. coli*	4981	1 (6%)	No	No	No	Yes	No	No	No	No	No	No	Yes	Yes	No	No	No	No	Yes	Yes	No	No	No	No	No	Yes	No	No	IncF, col
SAMN47862005	*K. pneumoniae*	307	3 (27%)	No	No	Yes	No	No	No	No	No	No	No	Yes	Yes	No	Yes	No	Yes	Yes	Yes	Yes	No	No	No	Yes	No	No	No	IncF
SAMN47862013	*K. pneumoniae*	307		No	No	Yes	No	No	No	No	No	No	No	Yes	Yes	No	Yes	No	Yes	Yes	Yes	Yes	No	No	No	Yes	No	No	No	IncF
SAMN47862023	*K. pneumoniae*	307		No	No	Yes	No	No	No	No	No	No	No	Yes	Yes	No	Yes	No	Yes	Yes	Yes	Yes	No	No	No	Yes	No	No	No	IncF
SAMN47862019	*K. pneumoniae*	147	2 (18%)	No	Yes	No	No	No	No	No	No	No	No	Yes	Yes	No	Yes	Yes	No	No	No	Yes	Yes	Yes	Yes	Yes	No	Yes	No	IncF, col
SAMN47862020	*K. pneumoniae*	147		Yes	No	No	No	No	No	No	No	No	No	Yes	Yes	No	Yes	No	No	No	No	Yes	Yes	Yes	No	Yes	No	No	No	IncF, col
SAMN47862018	*K. pneumoniae*	323	2 (18%)	No	Yes	No	No	No	No	No	No	No	No	Yes	Yes	No	Yes	No	Yes	Yes	Yes	Yes	Yes	No	No	Yes	No	No	No	IncF, IncI
SAMN47862024	*K. pneumoniae*	323		No	Yes	No	No	No	No	No	No	No	No	Yes	Yes	No	Yes	No	Yes	Yes	Yes	Yes	No	No	No	Yes	No	No	No	IncF
SAMN47862006	*K. pneumoniae*	ND	2 (18%)	Yes	No	No	No	No	No	No	No	No	No	No	Yes	No	Yes	No	Yes	Yes	Yes	Yes	Yes	No	No	Yes	No	No	No	IncF, IncI, col
SAMN47862016	*K. pneumoniae*	ND		Yes	No	No	No	No	No	No	No	No	No	No	Yes	Yes	No	No	No	Yes	Yes	No	Yes	Yes	No	No	No	Yes	No	IncF, IncI, col
SAMN47862012	*K. pneumoniae*	101	1 (9%)	No	No	No	No	No	No	Yes	No	No	No	Yes	No	No	Yes	No	Yes	Yes	Yes	Yes	Yes	No	No	No	No	No	Yes	IncF, IncI, col
SAMN47862036	*K. pneumoniae*	25	1 (9%)	Yes	No	No	No	No	No	No	No	No	No	Yes	No	No	Yes	No	Yes	Yes	Yes	Yes	No	No	No	No	Yes	No	No	IncF, IncR, col
SAMN47862021	*A. baumannii*	642	1 (33%)	No	No	No	No	No	Yes	No	Yes	No	No	No	No	No	No	No	No	No	No	No	No	No	No	No	No	No	No	–^*b*^
SAMN47862026	*A. baumannii*	23	1 (33%)	No	No	No	No	No	Yes	No	No	No	No	No	No	No	No	No	No	No	No	No	No	No	No	No	No	No	No	IncF, IncI, IncX, col
SAMN47862017	*A. baumannii*	ND	1 (33%)	No	No	No	No	No	No	No	No	Yes	Yes	No	No	No	No	No	Yes	No	No	Yes	No	No	No	No	No	No	No	IncF, IncX, IncH
SAMN47862025	*P. mirabilis*	ND	2 (100%)	No	Yes	No	No	No	No	No	No	No	No	No	No	No	No	Yes	No	No	Yes	No	Yes	No	Yes	No	No	Yes	No	–
SAMN47862037	*P. mirabilis*	ND		No	Yes	No	No	No	No	No	No	No	No	No	No	No	No	Yes	No	Yes	Yes	No	Yes	No	Yes	No	No	Yes	No	–
SAMN47862008	*E. hormaechei*	171	1 (50%)	No	No	No	Yes	No	No	No	No	No	No	Yes	No	No	No	No	No	No	No	No	No	No	No	No	Yes	No	No	IncF, col
SAMN47862009	*E. hormaechei*	121	1 (50%)	No	No	No	Yes	No	No	No	No	No	No	No	Yes	No	No	No	Yes	Yes	Yes	Yes	No	No	No	Yes	No	No	No	IncF, col
Number				**15**	**5**	**3**	**7**	**4**	**2**	**1**	**1**	**1**	**1**	**21**	**20**	**13**	**10**	**3**	**17**	**17**	**16**	**15**	**15**	**5**	**3**	**10**	**9**	**5**	**4**	
Percentage (%)				**42.9**	**14.3**	**8.6**	**20.0**	**11.4**	**5.7**	**2.9**	**2.9**	**2.9**	**2.9**	**60.0**	**57.1**	**37.1**	**28.6**	**8.6**	**48.6**	**48.6**	**45.7**	**42.9**	**42.9**	**14.3**	**8.6**	**28.6**	**25.7**	**14.3**	**11.4**	

^*a*^ Yes indicates present, no indicates absent.

^*b*^ "–" indicates no plasmid replicon detected.

^*c*^ Gray shading indicates the presence of the corresponding gene.

### Resistomes of the isolates

Of the strains studied, *bla_NDM-5_* was the most frequently detected carbapenemase gene (15/35; 42.9%), identified in *E. coli* (ST648, ST410, ST405, ST361, and ST90), as well as in *K. pneumoniae* (ST25 and ST147). The *bla_NDM-1_* gene was detected (5/35; 14.3%) in *K. pneumoniae* (ST323 and ST147) and *Proteus mirabilis*. The *bla_OXA-181_* gene was detected (7/35; 20.0%) in *E. coli* (ST10, ST44, ST410, and ST4981) and *Enterobacter hormaechei* (ST121 and ST171). The *bla_OXA-484_* gene was also identified (4/35; 11.4%), exclusively in *E. coli* (ST410 and ST167) (Table 3). Co-occurrence of carbapenemase genes was observed (6/35; 17.1%), including *bla_NDM-5_ + blaOXA_OXA-181_* (3/35; 8.6%) in *E. coli* (ST410 and ST44), as well as *bla_OXA-23_ + bla_OXA-68_* (2/35; 5.7%) and *bla_OXA-70_ + blaOXA_OXA-420_* (1/35; 2.9%), exclusively in *A. baumannii*.

Among the isolates studied*, bla*_*CTX-M-15*_ (21/35, 60%) was the most frequently associated ESBL with carbapenemases, found in the majority of *E. coli* (ST410, ST648, ST361, ST4981, ST44), *K. pneumoniae* (ST307, ST147, ST101, ST323, ST25), and *E. hormaechei* (ST171). Additionally, the bla*_VEB-6_* gene was identified in three isolates (3/35; 8.6%): one *K. pneumoniae* ST147 and two *P. mirabilis* strains.

Regarding aminoglycoside resistance genes, *aac(6′)-Ib-cr* (17/35, 48.6%) was widely distributed among *E. coli* (ST410, ST90, ST44), *K. pneumoniae* (ST307, ST101, ST323, ST25), *E. hormaechei* ST121, and *A. baumannii*. The methyltransferase *rmtB* (5/35, 14.3%), conferring high-level resistance to aminoglycosides, was detected in *E. coli* (ST410, ST90) and *K. pneumoniae* (ST147). Finally, *armA* (3/35; 8.6%) was found in two isolates: one *K. pneumoniae* ST147 and one *P. mirabilis* strain.

For quinolone resistance, plasmid-mediated genes *qnr*B*, qnr*S1, and *qnr*A were frequently co-present with carbapenemases. *qnr*B (10/35, 28.6%) was detected in *E. coli* ST410 and multiple *K. pneumoniae* isolates (ST307, ST147, ST323), as well as in *E. hormaechei* ST121. *qnr*S1 (9/35, 25.7%) was particularly observed in *E. coli* (ST410, ST90, ST4981, ST167, ST10), *K. pneumoniae* (ST25) and *E. hormaechei* (ST171). *qnr*A (5/35, 14.3%) was identified in *E. coli* ST410, *K. pneumoniae* (ST147), and *P. mirabilis* (Table 3).

The most frequently detected plasmid replicons were IncF (32/35, 91.43%), followed by Col (22/35, 62.86%), IncI (11/35, 31.43%), and IncX (2/35, 5.71%). Multiple replicons were identified simultaneously in some isolates (Table 3).

## DISCUSSION

Carbapenem resistance among Gram-negative bacilli represents a growing global health threat due to their high potential to cause severe, difficult-to-treat infections and their capacity for rapid dissemination via mobile genetic elements (1). This issue affects many countries, particularly in Africa, where data remain limited (14). The present study aimed to characterize resistomes of carbapenem-resistant bacteria isolated in Senegal using whole-genome sequencing analyses.

In this study, the majority of patients were male (54.29%). More than half of the patients (62.86%) were hospitalized, suggesting a possibility of nosocomial transmission of these bacteria, as reported in the literature (15). The most frequent specimen types were blood cultures (31%), pus (29%), and urine (26%). These findings are consistent with several studies showing that infections caused by carbapenem-resistant *Enterobacteriaceae* often occur in hospitalized patients from wounds, blood, and urine (14, 16). These data underscore the importance of preventive measures, such as hand hygiene, surface cleaning, and isolation of carrier patients.

Our results demonstrated very high resistance to β-lactams, including third-generation cephalosporins (>96%) and carbapenems (>96%), as well as to fluoroquinolones (100%), significantly limiting therapeutic options. The multidrug resistance is clinically concerning, as it complicates the management of severe infections, particularly in hospital settings. Similar findings have been reported in Senegal and in other Asian countries (China and South Korea), where carbapenem-resistant *Enterobacteriaceae* frequently exhibited co-resistance to major antibiotic classes (17–19).

In this context, our data highlight the relevance of aminoglycosides, particularly amikacin, which retain good activity with relatively low resistance rates (23%). Species-specific analysis confirms this trend, with 100% susceptibility in *Acinetobacter baumannii* (3/3) and *Enterobacter hormaechei* (2/2), 76% in *Escherichia coli* (13/17), 73% in *Klebsiella pneumoniae* (8/11), and 50% in *Proteus mirabilis* (1/2). These findings are consistent with data reported in the literature (14, 20) and further support the potential therapeutic value of amikacin. This relatively promising susceptibility, also reported in other studies (19), positions amikacin as a viable therapeutic alternative, particularly in combination therapy or targeted treatment based on antibiogram results. These findings emphasize the need for rational adaptation of treatment protocols according to local resistance profiles in order to preserve the efficacy of the few remaining active antibiotics.

The results of this study confirm the presence, in our hospital setting, of major carbapenemases mainly belonging to the *NDM* family (23; 23/35) and *OXA-type* (18; 18/35) carbapenemases, identified in *E. coli* (48%), *K. pneumoniae* (31%), *A. baumannii* (9%), *Proteus mirabilis* (6%), and *Enterobacter hormaechei* (6%). These mechanisms were associated with clonal lineages recognized for their international dissemination, such as *E. coli* ST410, as well as *K. pneumoniae* ST147 and ST307 (21). The *bla_NDM-5_, bla_NDM-1_, and bla_OXA-181_* genes identified in our series have previously been reported in Senegal and several West African countries, suggesting regional circulation of these determinants (22, 23). The co-occurrence of carbapenemases, including the *bla_NDM-5_ + blaOXA_OXA-181_* association and the combinations of OXA enzymes observed in *A. baumannii* (*OXA-23* with *OXA-68* and *OXA-70* with *OXA-420*), highlights the complexity of resistance profiles observed in our collection of isolates and may further limit therapeutic options while complicating the clinical management of infections caused by these multidrug-resistant isolates.

Molecular analysis confirmed the phenotypic multidrug-resistance profiles observed, with a high prevalence of the *bla*_*CTX-M-15*_ gene (21/35), consistent with the elevated resistance rates to third-generation cephalosporins. The predominance and persistence of this gene in this hospital and globally have been previously reported (13, 24). To our knowledge, this study reports for the first time in Senegal the detection of the *bla_VEB-6_* gene. This gene confers high-level resistance to third-generation cephalosporins, including ceftazidime, cefotaxime, and aztreonam, and has been sporadically reported in Europe, notably in Italy and Switzerland (25, 26). It is generally located within integrons, which facilitates its horizontal dissemination and association with other resistance determinants (26). In our study, *bla_VEB-6_* was detected in co-occurrence with genes conferring high-level resistance to aminoglycosides, such as *rmtB* and *armA*, which is particularly concerning because this combination severely limits available therapeutic options, especially amikacin. Other genes, such as *aac(6′)-Ib-cr, aadA, aph(3″)-Ib*, as well as plasmid-mediated quinolone resistance genes (*qnrA, qnr*B*, qnr*S1), were also detected. These genetic profiles reflect a co-resistance dynamic similar to that reported in other countries, such as Jordan and Angola, highlighting the emergence of high-risk clones in our local context (27, 28). The accumulation of these genes within the same strain illustrates the growing threat posed by multidrug-resistant GNB in African hospital settings and underscores the need for continuous genomic surveillance and enhanced control strategies.

Genomic analysis also revealed the presence of multiple plasmid groups, including IncF, IncX3, IncI, and Col, known for their capacity to carry multiple resistance genes (29, 30). These mobile genetic elements play a central role in the horizontal dissemination of genes, such as *bla*_*CTX-M-15*_, *bla*_*NDM*_, *rmt*B*, arm*A, and *qnr* (29). The IncF type, particularly frequent in our study, is well documented for its ability to simultaneously carry ESBL, carbapenemase, aminoglycoside, and fluoroquinolone resistance genes (31). These findings highlight the importance of monitoring not only resistance genes but also the genetic vectors that ensure their transmission and stability within hospital bacterial populations.

MLST analysis revealed significant clonal diversity, dominated by high-risk epidemiological clones. In *E. coli*, ST410 (*n* = 7), carrying *bla*_*NDM-5*_ and *bla_OXA-48-like_* carbapenemases (*bla*_*OXA-181*_, *bla*_*OXA-484*_), as well as ST648 (*n* = 3) with bla*_NDM-5_*, were the most frequent. In *K. pneumoniae*, ST307 (*n* = 3, *bla*_*NDM-15*_) and ST147 (*n* = 2, *bla*_*NDM-1*_ and *bla*_*NDM-5*_) predominated. These clones are well documented in the literature for their role in the global dissemination of carbapenemases and their involvement in nosocomial outbreaks (21). ST410 is an emerging *E. coli* clone reported in Africa and in many regions worldwide (10, 21), whereas ST307 and ST147 are recognized as high-risk *K. pneumoniae* clones circulating in South Korea (32), Germany (33), and Africa (21). These data place the strains from our study within a global epidemiological context, highlighting the need for continuous and coordinated genomic surveillance, particularly in hospital settings where these clones can spread rapidly.

### Limitations of this study

Although this study provided relevant data on antimicrobial resistance (AMR) in West Africa, several limitations should be noted. The limited number of samples analyzed and the fact that the isolates originated from a single hospital center limit the representativeness of the results and prevent their generalization to the entire population. Some clinical and epidemiological information was unavailable, including travel history and the distinction between colonization and infection, limiting the assessment of risk factors and modes of transmission. At the molecular level, we did not characterize the complete plasmid genomes or search for virulence genes, which would have provided a better understanding of AMR dissemination and its clinical impact. Finally, phylogenetic analyses could have clarified the genetic relatedness with other strains reported internationally. Although these limitations restrict the interpretation of the results, they highlight the need to strengthen microbiological surveillance and infection prevention measures. These points will be addressed in future studies.

### Conclusion

This study highlights the presence of multidrug-resistant Gram-negative bacilli in a Senegalese hospital, as well as the detection of key resistance genes, such as *bla*_*NDM-1*_*, bla*_*NDM-5*_*, bla*_*CTX-M-15,*_ *bla*_*OXA-181*_, and, for the first time in Senegal, the *bla*_*VEB-6*_ gene. The coexistence of multiple plasmid types within the same isolates suggests the great involvement of MGEs in the dissemination of AMR in Senegal. The identification of high-risk clones, such as ST410 and ST648 in *E. coli* and ST307 and ST147 in *K. pneumoniae*, underscores the urgent need to strengthen genomic surveillance and implement strict infection prevention and control measures. These findings also provide a foundation for future research on plasmid transfer and co-resistance, which are essential to limit the spread of these multidrug-resistant strains.

## MATERIALS AND METHODS

### Bacterial identification and antimicrobial susceptibility testing

This retrospective study was conducted at the Centre Hospitalier National Universitaire (CHNU) de Fann, a level III tertiary-care hospital and reference center located in Dakar, which manages both hospitalized patients and outpatients and includes several specialized departments (notably neurology, neurosurgery, and cardiology). During the study period (October 2022 to March 2024), 133 Gram-negative bacilli showing resistance to ertapenem (inhibition zone diameter ≤25 mm) and/or imipenem (inhibition zone diameter ≤22 mm) were isolated and considered suspected carbapenemase producers, according to CA-SFM criteria (2022–2023) (34, 35). These isolates were initially stored at −80°C in brain-heart infusion broth for subsequent molecular analysis. The final inclusion in the study was contingent upon the viability of the strains after storage; consequently, isolates that did not survive the cold storage process were excluded from further analysis. Among the 133 initially identified isolates, 50 could be recovered after storage and were retained for whole-genome sequencing. Initial identification was performed in the bacteriology laboratory of CHNU de Fann using the automated Vitek system and subsequently confirmed by MALDI-TOF mass spectrometry at the Institut Pasteur de Dakar. Antimicrobial susceptibility testing was performed using the disk diffusion method, in accordance with CA-SFM guidelines applicable at the time of the study (2022–2023) (34, 35). Finally, among the 50 sequenced isolates, 35 were confirmed as carbapenemase producers and selected for the in-depth genomic analyses presented in this study.

### DNA extraction

Bacterial DNA was extracted using the QIAamp DNA Mini Kit (Qiagen, Hilden, Germany), following the manufacturer’s instructions. Samples were subjected to lysis with proteinase K, followed by incubation at 56°C for 1–2 h to optimize DNA release. Purification was performed using silica columns, with successive centrifugation steps to remove impurities. The purified DNA was eluted in Buffer AE and quantified using a Qubit fluorometer. The resulting extracts were subsequently used for whole-genome sequencing.

### Whole-genome sequencing

Sequencing was performed on the Illumina NextSeq 500 platform using a 300-cycle cartridge, generating 2 × 150 bp paired-end reads (PE150). Library preparation was conducted with the Illumina DNA Prep (DNA Flex) kit, which included DNA fragmentation via tagmentation, followed by PCR amplification with specific indices. The PCR protocol consisted of an initial denaturation at 98°C, followed by six amplification cycles (98°C, 30 s; 62°C, 30 s; 68°C, 2 min) and a final extension at 68°C for 1 min. Amplified products were purified, resuspended, normalized, and pooled at equimolar concentrations prior to sequencing.

### Bioinformatic analyses

Raw reads were assessed using FastQC (36) and subsequently cleaned with fastp (37) to remove low-quality bases and adapter sequences. *De novo* assembly was performed with SPAdes (38) and evaluated using QUAST (39). Taxonomic identification was carried out with MASH (40), followed by decontamination using Kraken2 and KrakenTools (41). Genome completeness was verified with CheckM2. Antibiotic resistance genes and plasmid replicons were detected using Abricate (42) with the ResFinder and PlasmidFinder databases (threshold <98%) (43). Sequence types (MLST) were determined from the assembled genomes, allowing reliable identification of STs for each strain (42).

### Statistical analysis

Data were entered and analyzed using Microsoft Excel 2024 (LTSC version, Microsoft Corporation). Qualitative variables, such as bacterial species, presence of resistance genes, and antibiotic resistance profiles, are expressed as frequencies and percentages and presented in tables. No sample size calculation or advanced statistical tests were performed.

## Data Availability

A total of 35 isolates carrying at least one carbapenem resistance gene have been deposited in the NCBI database under the BioProject accession number PRJNA1248572.
